# Recurrent Cardiac Tamponade from Multiple Myeloma While Receiving Teclistamab

**DOI:** 10.1016/j.jaccas.2024.102763

**Published:** 2024-12-04

**Authors:** Alexander Hurtado, Samuel T. Luebbe, Emma Kilbane, Toluwalase Awoyemi, Taylor Bronson, Jayesh Mehta, Mohamed M.H. Al-Kazaz, Adam Y. Lin

**Affiliations:** a McGaw Medical Center of Northwestern University, Chicago, Illinois, USA; b Department of Pathology, Feinberg School of Medicine, Northwestern University, Chicago, Illinois, USA; c Division of Hematology and Oncology, Department of Medicine, Feinberg School of Medicine, Northwestern University, Chicago, Illinois, USA; d Robert H. Lurie Comprehensive Cancer Center of Northwestern University, Chicago, Illinois, USA; e Division of Cardiology, Department of Medicine, Feinberg School of Medicine, Northwestern University, Chicago, Illinois, USA; f Bluhm Cardiovascular Institute of Northwestern, Chicago, Illinois, USA

**Keywords:** cardiac tamponade, pericardial effusion, teclistamab

## Abstract

Bispecific therapy has changed the treatment paradigm for multiple myeloma. We report a patient with recurrent malignant pericardial effusions with cardiac tamponade and new atrial fibrillation during treatment, suggesting that new or worsening pericardial disease may be a potential cardiovascular adverse effect of bispecific therapy.

## Graphical Abstract

**Figure:**
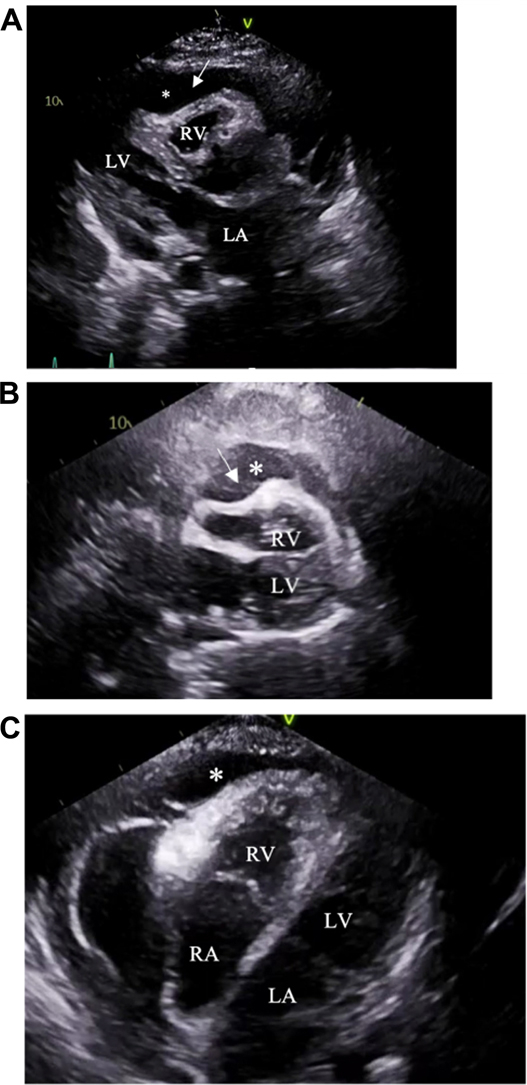

## History of Presentation

Patient X is a 55-year-old woman with high-risk immunoglobulin A lambda multiple myeloma (deletion 17p, +1q, [t14;16]). She underwent induction therapy with daratumumab, lenalidomide, bortezomib, and dexamethasone with a very good partial response. She then had an autologous hematopoietic stem cell transplant with high-dose melphalan. Unfortunately, restaging laboratory work and imaging demonstrated progressive disease. Therefore, she was initiated on teclistamab for relapsed refractory multiple myeloma. The patient received the first step-up dose of teclistamab without any adverse effects of the bispecific therapy. Forty-eight hours later, she received the second step-up dose. The next day, she developed a fever with elevating ferritin and c-reactive protein. Later that day, the patient required urgent transfer to the cardiac critical care unit in shock despite fluid resuscitation with hypotension, dyspnea, and chest discomfort. Tocilizumab was given with initial concern for cytokine release syndrome (CRS) as the etiology of hypotension and tachycardia without clinical improvement. The physical examination was remarkable for a blood pressure of 73/51; heart rates of up to 120 beats/min, notable jugular venous distention, and attenuated heart sounds.

**Take-Home Messages**

• Bispecific antibody therapies may have under-reported cardiovascular adverse events.
• Rare causes of hypotension, such as cardiac tamponade, should be considered early in patients with pericardial involvement of disease when undergoing bispecific antibody therapy.
• Treatment for recurrent malignant pericardial effusion includes pericardiocentesis initially, followed by pericardial window and, if indicated, pericardial radiation.

## Differential Diagnosis

Differential diagnoses for the patient’s initial presentation of shock were broad, including cardiogenic, septic, obstructive, and CRS related to bispecific antibody therapy.

## Investigations

A bedside ultrasound was conducted, which showed circumferential pericardial effusion and right ventricular collapse. Electrocardiography showed low-voltage QRS complex in all leads. It was determined that the patient was experiencing obstructive shock caused by cardiac tamponade. Infectious evaluation was negative.

## Management

**Figure 1:**
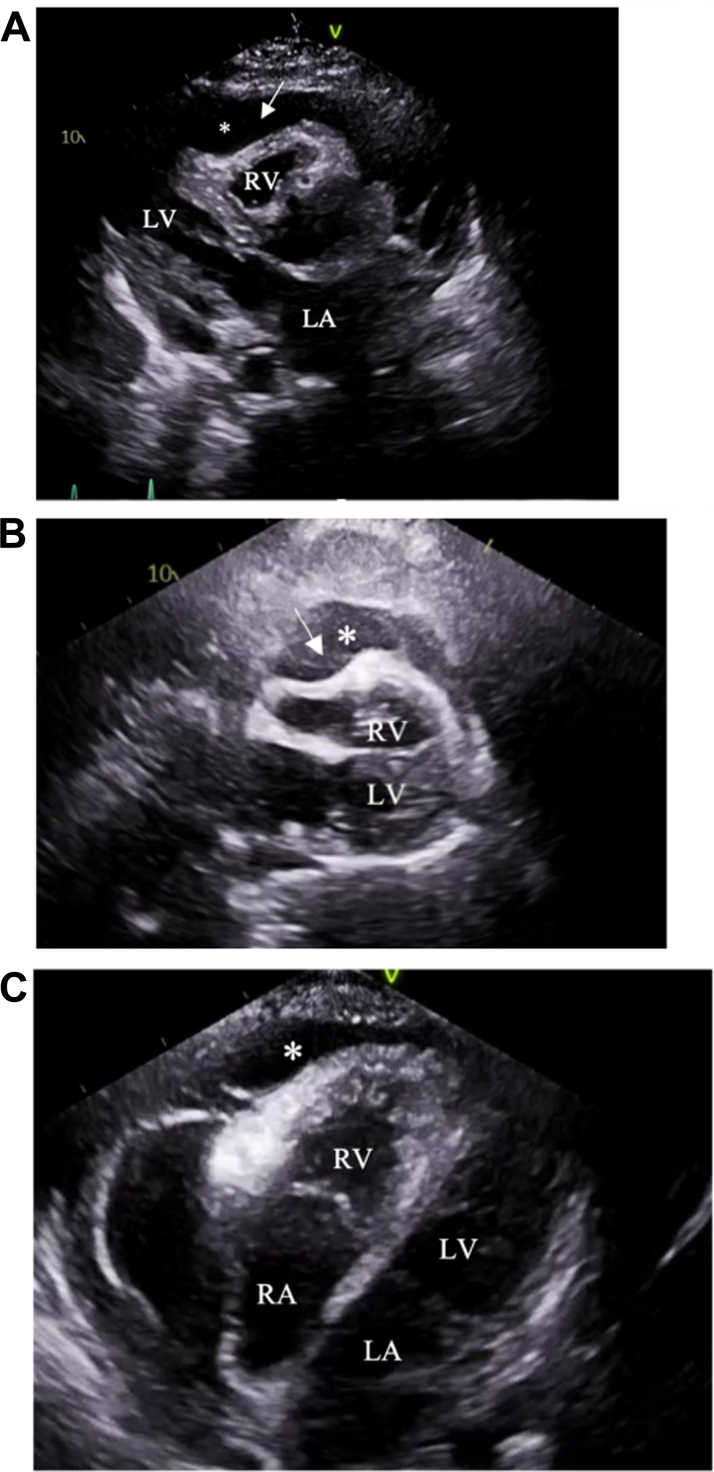
Transthoracic Echocardiography Showing Recurrent Pericardial Effusion and Tamponade (A) Parasternal long-axis view demonstrating large pericardial effusion and right ventricular (RV) collapse. (B) Subxiphoid view demonstrating large pericardial effusion and RV collapse. (C) Apical 4-chamber view with off-axis orientation demonstrating large pericardial effusion. Arrow indicates RV collapse. ∗Pericardial effusion. LA = left atrium; LV = left ventricle; RA = right atrium.

The patient underwent emergent pericardiocentesis with pericardial drain placement and fluid analysis (Supplemental Figure 1), which demonstrated atypical plasma cells with concern for myelomatous extension into the pericardial space. She was subsequently initiated on colchicine to mitigate concurrent pericardial inflammation. Serial transthoracic echocardiography showed gradual improvement in the effusion, prompting the removal of the pericardial drain 2 days post-placement (Video 1, Video 2). Pericardial drain output consisted of 230 mL for the first 24 hours, decreasing to 15 mL 24 hours before the day of removal. Two days postdrain removal, the patient developed tachycardia (160 beats/min) with reaccumulation of a large pericardial effusion. There was concern for impending cardiac tamponade physiology. Therefore, she had a video-assisted thoracoscopic construction of a pericardial window with a pericardial biopsy (Supplemental Table 1). Pericardial biopsy showed fibrinous pericarditis. Following window creation, the patient received the first full dose of teclistamab, which was delayed by 6 days. Despite window creation, the patient had pericardial effusion reaccumulation (Figures 1A to 1C, Video 3, Video 4, Video 5, Video 6, Video 7, Video 8, Video 9, Supplemental Figures 2 and 3), requiring another pericardial drain followed by 2 fractions of radiation (2Gy) to the pericardium. Pericardial drain output and duration of placement (10 days) were increased compared with the initial drain, with a total withdrawal amount of nearly 2 L of fluid. On day 10, pericardial drain output was about 5 mL, and a transthoracic echocardiogram confirmed a reduction in pericardial effusion size (Videos 10 and 11), leading to drain removal.

Following the second drain removal, the patient’s pericardial effusion did not recur. However, her course was further complicated by multiple episodes of new atrial fibrillation with rapid ventricular response after receiving the third full dose of teclistamab. The patient underwent cardioversion with a return to normal sinus rhythm and was initiated on amiodarone for ongoing rhythm control. The patient continued receiving weekly teclistamab for a total of 2 cycles without experiencing any further cardiac events. Eight weeks after the initial diagnosis of cardiac tamponade secondary to malignant pericardial effusion, the patient achieved a complete remission. She underwent a haploidentical allogeneic stem cell transplant and was able to return to her normal life while receiving teclistamab maintenance. Unfortunately, the patient ended up relapsing in the central nervous system space and was transitioned to comfort care before the fabrication of chimeric antigen receptor T cells was able to be completed.

## Discussion

The prevalence of extramedullary plasma-cell infiltration in multiple myeloma to the pericardium is uncommon.^1^ Plasma cell infiltration may lead to pericardial effusion that has the potential to progress to cardiac tamponade, which is a very rare occurrence. Treatment for cardiac tamponade, in the immediate phase, consists of pericardiocentesis.^2^ However, with reaccumulation, a pericardial window between the pericardium and the pleura or peritoneum is often needed, along with systemic chemotherapy and/or radiation in malignant effusions.^2^^,^^3^

**Figure 2:**
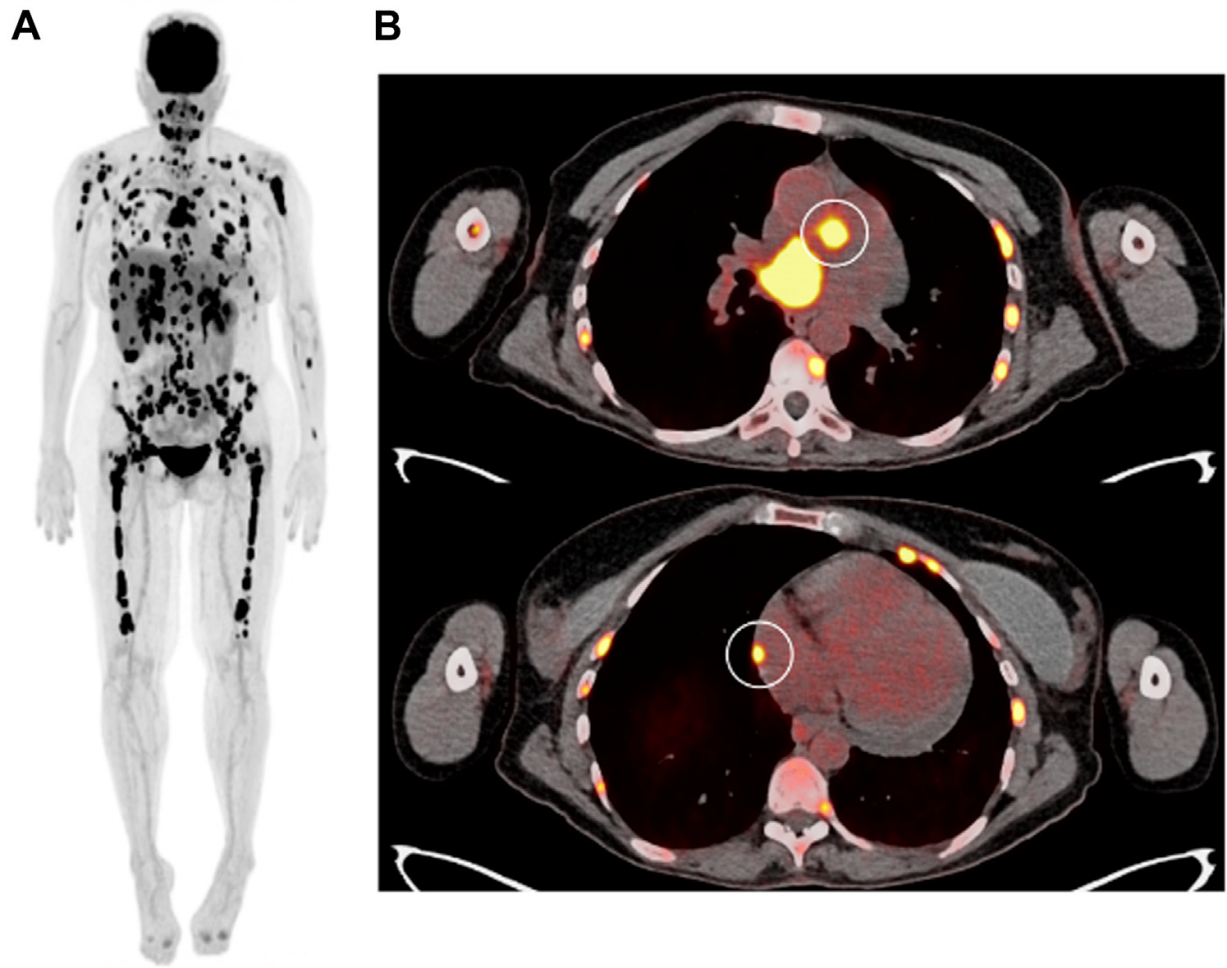
Fludeoxyglucose Positron Emission Tomography Scan for the Patient Before Starting Bispecific Therapy (A) A whole-body positron emission tomography scan showed extensive disease in several involved nodes and organs in the chest. The dark areas are possible myeloma involvement, excluding the bladder and brain, which are always positive. (B) Positron emission tomography avid lesions involving the pericardium are identified in the white circles.

In this case report, we share a rare occurrence of recurrent malignant pericardial effusions and cardiac tamponade while undergoing multiple myeloma treatment with teclistamab. Teclistamab is a U.S. Food and Drug Administration–approved bispecific antibody that targets B-cell maturation antigen and CD3 to treat relapsed or refractory multiple myeloma.^4^ The etiology of recurrent pericardial effusion and subsequent cardiac tamponade may be secondary to pericardial infiltration of multiple myeloma before treatment, because there was patchy hypermetabolic activity along the right lateral pericardium and pericardial recess on a pretreatment positron emission tomography-computed tomography (PET) scan (Figure 2). However, there was pericardial disease stability over the time duration (1 month) between PET acquisition and the time of admission for teclistamab initiation. Only after treatment was initiated with bispecific therapy the pericardial effusion accumulation rapidly accelerated, leading to recurrence of cardiac tamponade despite pericardial window creation. The increased rate of pericardial effusion accumulation that coincided with teclistamab initiation primes the consideration of 2 more etiologies: pseudoprogression and potential cardiovascular adverse effect of teclistamab.

Pseudoprogression is characterized by an initial increase in cancer burden following the initiation of immunotherapies, such as teclistamab, followed by a spontaneous decrease in disease burden.^5^ Therefore, given that the patient had pericardial disease observed on PET that was previously stable, it is possible that the rapid acceleration of pericardial effusion and subsequent tamponade was caused by pseudoprogression. However, the improvement in disease burden in the pericardium was not spontaneous and required multiple interventions, including repeat pericardiocentesis, pericardial window, and radiation, to achieve significant improvement, which may point away from this etiology.

Alternatively, the recurrent pericardial effusion may represent an under-reported adverse effect of teclistamab therapy. Previously documented adverse effects from teclistamab include CRS and immune cell-associated neurotoxicity syndrome.^4^ However, the patient received Tocilizumab, cornerstone therapy for CRS, during initial concern for CRS without any clinical response. Therefore, we may reason that there may be under-reported cardiovascular adverse effects from teclistamab independent of CRS. Cardiovascular adverse events (CVAEs) that have been observed early in the administration of teclistamab step-up doses included myocarditis (7%), unspecified shock (20%), atrial fibrillation (6%), and heart failure (6%).^6^ Furthermore, other bispecific T-cell engager therapies with similar mechanisms of action, such as blinatumomab, have been associated with CVAEs including pericardial effusion, though rare (<0.5%).^7^ Further investigation into CVAEs of teclistamab during the step-up dosing period, as well as later in treatment cycles, is needed to evaluate this medication as an etiology of pericardial effusion and cardiac tamponade.

Cases of multiple myeloma with pericardial involvement are very rarely reported and have a poor prognosis with a median survival of only 6 weeks postdiagnosis of malignant pericardial effusion.^1^ In this case, we observe that these bispecific antibody therapies may provide an opportunity to achieve complete remission and a chance of cure with allogeneic stem cell transplant for the traditionally grim prognosis that would typically be expected in cases of pericardial infiltration of multiple myeloma. Although it is possible that teclistamab played a significant role in the development and recurrence of the pericardial effusion and tamponade physiology in this patient, it also was critical to her ultimate remission. It may be possible that the pericardial effusion represented a positive response to treatment, as in the case of pseudoprogression; however, we cannot exclude that the addition of bispecific antibody therapies may lead to CVAEs.

## Conclusions

Teclistamab has provided life-saving treatment for our patients and many others with multiple myeloma. However, this case raises questions about whether preventative monitoring for CVAEs should be considered in bispecific antibody therapies, such as fixed interval echocardiography, or if prophylactic radiation should be used for patients with known pericardial involvement.

## Funding Support and Author Disclosures

Dr Al-Kazaz has received a research grant and speaker honoraria from Kiniksa Pharmaceuticals. Dr Lin has received speaker honoraria from Abbvie and Genmab. All other authors have reported that they have no relationships relevant to the contents of this paper to disclose.

## Appendix

Video 1 Parasternal Long-Axis View of Transthoracic Echocardiography With Improving Pericardial Effusion With the First Pericardial Drain in Place

Video 2 Apical 4-Chamber View of Transthoracic Echocardiography With Improving Pericardial Effusion With the First Pericardial Drain in Place

Video 3 Transthoracic Echocardiogram Parasternal Long-Axis View After Pericardial Window Creation and First Full Dose of Teclistamab Showing Large Circumferential Pericardial Effusion and Right Ventricular Collapse

Video 4 Transthoracic Echocardiography in Apical 4-Chamber View Showing Large Circumferential Pericardial Effusion and Right Atrial Collapse

Video 5 Transthoracic Echocardiography of Cardiac Tamponade in Parasternal Short-Axis View With Aortic Valve in View

Video 6 Transthoracic Echocardiography of Cardiac Tamponade in Subcostal View

Video 7 Transthoracic Echocardiography of Cardiac Tamponade in Subcostal View of Inferior Vena Cava

Video 8 Transthoracic Echocardiography of Cardiac Tamponade in Apical 4-Chamber View With Color Doppler Across Tricuspid Valve

Video 9 Transthoracic Echocardiography of Cardiac Tamponade in Parasternal Short-Axis View With Color Doppler Across Tricuspid Valve

Video 10 Transthoracic Echocardiography With Improving Pericardial Effusion With the Second Pericardial Drain in Place With Parasternal Long-Axis View

Video 11 Transthoracic Echocardiography With Improving Pericardial Effusion With the Second Pericardial Drain in Place With Apical 4-Chamber View
