# Supplementary material for: Recurrent Cardiac Tamponade from Multiple Myeloma While Receiving Teclistamab
Source: JACC Case Rep. 2024 Dec 4;29(23):102763. doi: 10.1016/j.jaccas.2024.102763 (PMC11646921; doi:10.1016/j.jaccas.2024.102763)
Supplement: Supplemental Figures 1-3 and Supplemental Table 1 [file mmc12.docx]

**
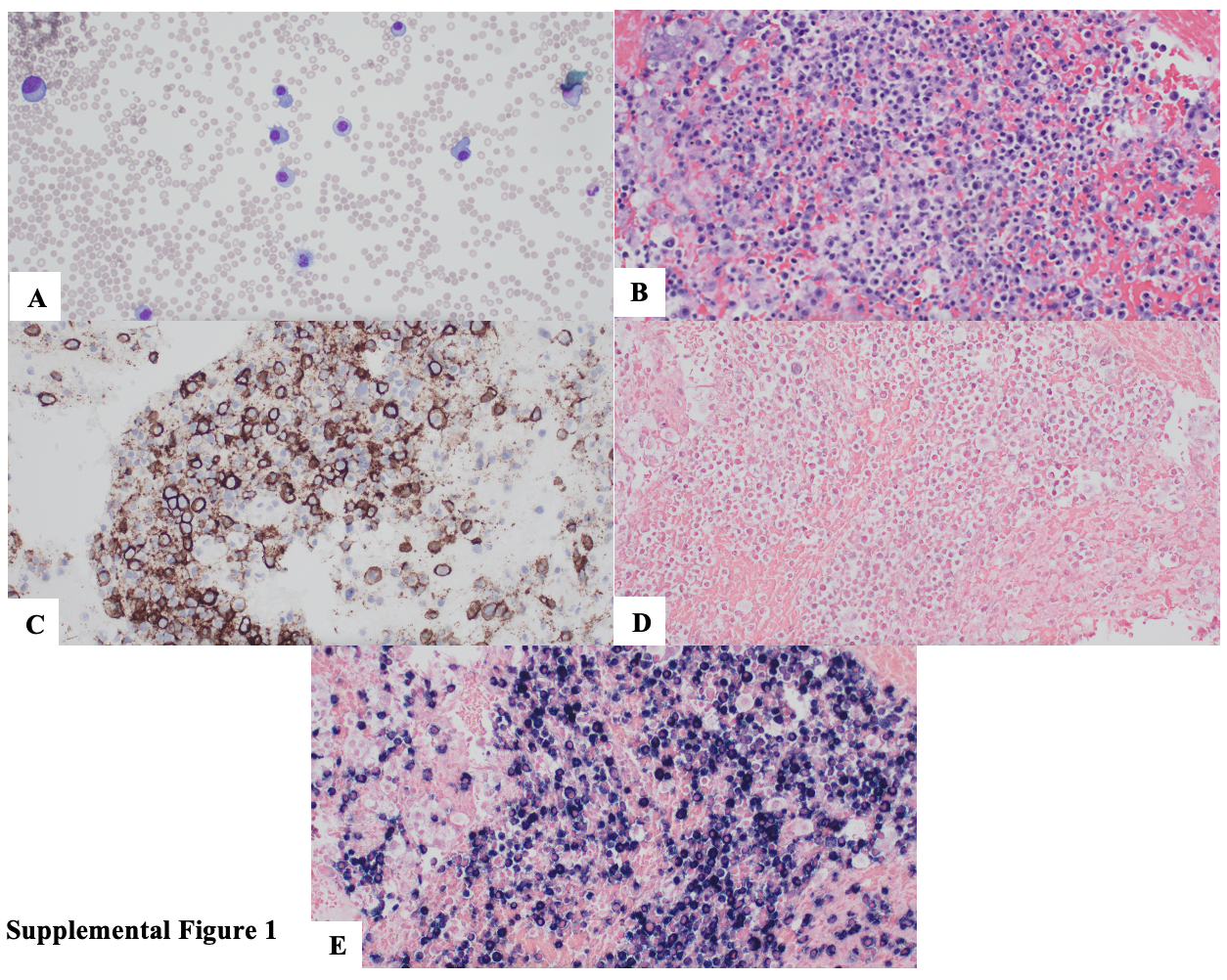
**

**Supplemental Figure 1: Pericardiocentesis fluid cytology, immunohistochemistry, and in-situ hybridization analysis.** (A) Wright-Giemsa Stain at 20x magnification showing rare atypical plasma cells, rare mesothelial cells, lymphocytes, and abundant polymorphonuclear leukocytes (B) Hematoxylin and eosin (H&E) stain at 20x magnification showing rare atypical plasma cells, neutrophils, lymphocytes, and rare mesothelial cells (C) CD138 stain at 20x magnification highlighting numerous atypical plasma cells (D) Chromogenic In-situ Hybridization (CISH) stain at 20x magnification for kappa light chain which is negative (E) CISH stain at 20x magnification for lambda light chain demonstrating that the atypical plasma cell population shows lambda light chain restriction with a lambda to kappa ratio greater than 100:1.

**
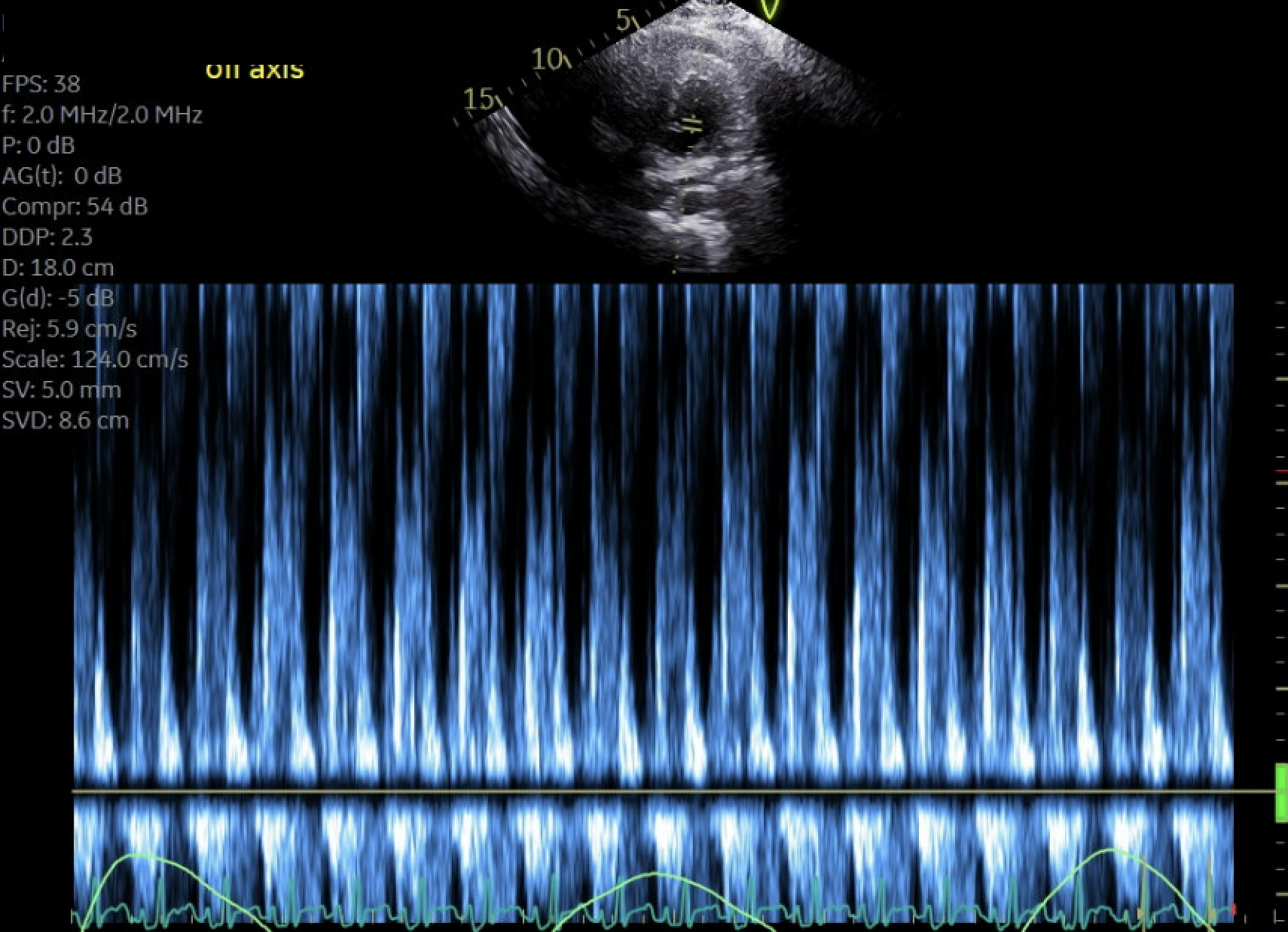
**

**Supplemental Figure 2: Mitral valve doppler acquired in apical 4-chamber view during cardiac tamponade.**

**
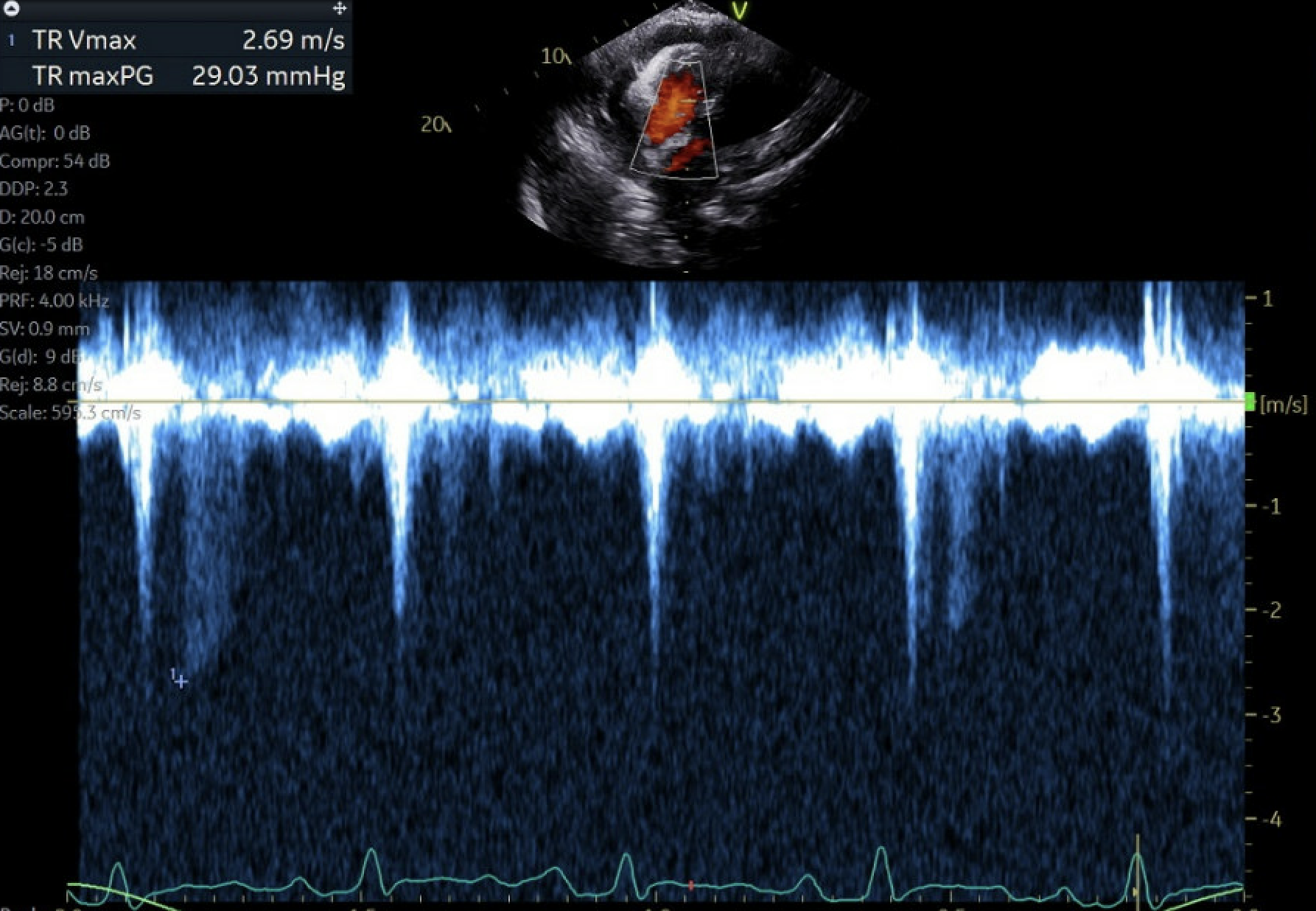
**

**Supplemental Figure 3: Tricuspid valve doppler acquired in apical 4-chamber view during cardiac tamponade**.

**Supplemental Table 1: Pericardial Tissue Biopsy Acquired During VATS Procedure.**

| **Microscopy Description:** | Sections demonstrate fibrinous pericarditis.  There is a mild chronic inflammation.  CD138 staining was performed and demonstrates a paucity of plasma cells.  Lambda and Kappa staining is also performed and demonstrates only background staining. The positive controls demonstrate appropriate positive staining.  The known tissue negative controls were negative.  The non-immune serum control was non-reactive |
| --- | --- |
| **Final Diagnosis:** | Fibrinous pericarditis |
